# Emergence of *Ixodes scapularis* and *Borrelia burgdorferi*, the Lyme disease vector and agent, in Ohio

**DOI:** 10.3389/fcimb.2014.00070

**Published:** 2014-06-04

**Authors:** Peng Wang, Meaghan N. Glowacki, Armando E. Hoet, Glen R. Needham, Kathleen A. Smith, Richard E. Gary, Xin Li

**Affiliations:** ^1^Department of Veterinary Biosciences, The Ohio State University, Columbus, OH, USA; ^2^Department of Veterinary Preventive Medicine, The Ohio State University, Columbus, OH, USA; ^3^Division of Epidemiology, The Ohio State University, Columbus, OH, USA; ^4^Department of Entomology, The Ohio State University, Columbus, OH, USA; ^5^Ohio Department of Health, Columbus, OH, USA

**Keywords:** Lyme disease, Ohio, *Borrelia burgdorferi*, *Ixodes scapularis*, *Peromyscus leucopus*

## Abstract

Lyme disease, the most common vector-borne disease in the United States, is caused by a tick-borne infection with *Borrelia burgdorferi*. Currently, Ohio is considered by the Centers for Disease Control and Prevention (CDC) to be non-endemic for Lyme disease. The low incidence of Lyme disease in this state was largely attributed to the absence of the transmitting vector, *Ixodes scapularis*, commonly known as the blacklegged tick. However, a tick surveillance program established by Ohio Department of Health indicated that the number of *I. scapularis* in Ohio had increased sharply in recent years, from 0 - 5 ticks per year during 1983–2008 to 15 in 2009, 40 in 2010, and 184 in 2011. During the fall deer hunting season, examination of deer heads submitted to Ohio Department of Agriculture found 29 *I. scapularis* from 7 counties in 2010 and 1,830 from 25 counties in 2011. As of 2012, the tick had been found in 57 of the 88 counties of Ohio. In addition, all three active stages (larva, nymph, and adult) of *I. scapularis* were found in Tiverton Township of Coshocton County, demonstrating the presence of established tick populations at this central Ohio location. Of 530 nymphal or adult *I. scapularis* analyzed by quantitative polymerase chain reaction (qPCR), 32 (6.1%) tested positive for the *B. burgdorferi flaB* gene, ranging from 36 to 390,000 copies per tick. Antibodies to *B. burgdorferi* antigens were detected in 2 of 10 (20%) field-captured *Peromyscus leucopus* from Tiverton Township, and in 41 of 355 (11.5%) dogs residing in Ohio. Collectively, these data suggest that the enzootic life cycle of *B. burgdorferi* has become established in Ohio, which poses risk of Lyme disease to people and animals in the area.

## Introduction

Since its initial description in late 1970's (Steere et al., 1977), Lyme disease has been recognized as the most common vector-borne disease in temperate regions of the northern hemisphere (Steere, 2001; Stanek et al., 2012). The disease is caused by spirochetes belonging to the *Borrelia burgdorferi* sensu lato complex, which is maintained by an enzootic life cycle typically involving *Ixodes* species ticks and small vertebrate hosts (Piesman and Gern, 2004). In the United States, Lyme disease is highly endemic in two distinct regions, one in the Northeastern states and the other in the Upper Midwestern states (Orloski et al., 2000; Bacon et al., 2008). In these regions, the blacklegged tick *Ixodes scapularis* is the transmitting vector, and the white-footed mouse *Peromyscus leucopus* serves as a common reservoir host for the spirochete (Piesman and Gern, 2004). Lyme disease is also reported in the Western United States, where the western blacklegged tick *I. pacificus* is the transmitting vector and the dusky-footed wood rat *Neotoma fuscipes* and California kangaroo rat *Dipodomys californicus* are the main reservoir hosts (Brown and Lane, 1992).

Ohio is situated between the Northeastern and the Upper Midwestern Lyme disease-endemic regions of the US, and it historically had a low incidence of diagnosed Lyme disease. According to CDC data from 2007 to 2012, the annual incidences of Lyme disease in Ohio and the three surrounding states to its west, Michigan, Indiana, and Kentucky, were all <1 case per 100,000 people; the annual incidences in the two states to the east of Ohio were higher, ranging 4–8 cases per 100,000 people in West Virginia and 26–37 cases per 100,000 people in Pennsylvania, a highly endemic area. The low incidence of Lyme disease in Ohio was largely attributed to the absence of *I. scapularis* in the area (Dennis et al., 1998; Hoen et al., 2009; Rollend et al., 2013). Here, we showed that since 2009, there had been an exponential increase in the number of *I. scapularis* found in Ohio. To further assess the risk of Lyme disease in Ohio, we investigated if there were established *I. scapularis* populations in Ohio, if the ticks were infected with *B. burgdorferi*, and if white-footed mice and dogs in Ohio had been exposed to *B. burgdorferi*. Our data suggest that the enzootic life cycle of *B. burgdorferi* has become established in Ohio.

## Materials and methods

### Ohio department of health tick surveillance program

In 1983, Ohio Department of Health (ODH) began soliciting ticks from the general public, hospitals, physicians, and local health departments in an effort to determine the distribution and dynamics of pathogens in Ohio tick populations. This program of passive tick surveillance for all tick species continued through 2012 and was promoted through media, university extension fact sheets, health department newsletters, presentations and select mailings (Pretzman et al., 1990). Active surveillance to specifically search for *I. scapularis* began in 1986, which included examination of trapped rodents, flagging vegetation at suspect Lyme disease locations, and examination of deer brought to Ohio Department of Natural Resources (ODNR) deer check stations. Tick collection records and specimens were maintained at ODH. Due to a loss of funding, the ODH tick surveillance program was discontinued in 2013.

### Examination of deer heads for ticks

From 2002 to 2011, Ohio Department of Agriculture (ODA) worked with ODNR and ODH to conduct surveillance of chronic wasting disease (CWD) during the fall deer hunting season by examining hunter-harvested deer heads. These deer heads were also examined for ticks. This active surveillance for CWD was discontinued in 2012.

### Tick survey in tiverton township

From March to November of 2010, ticks were collected from Tiverton Township in Coshocton County, Ohio by two different methods, flagging ticks from vegetation and soliciting ticks from local residents who found and removed them from people or domestic animals. All ticks collected in the area were identified by Glen R. Needham to be either *I. scapularis* or *Dermacentor variabilis* based on morphology.

### Quantitative polymerase chain reaction (qPCR) for detection of *B. burgdorferi* DNA

DNA was purified from individual or pooled ticks using the DNeasy Blood and Tissue Kit (QIAGEN) following the manufacturer's protocol, and qPCR reactions (25-μl each) were set up in 96-well plates using TaqMan Universal PCR Master Mix (Applied Bioscience) and a pair of primers and a TaqMan probe specific for the *B. burgdorferi flaB* gene (Li et al., 2007). The usage of a sequence-specific fluorescent probe in qPCR analysis not only provides a higher level of specificity than conventional PCR but also allows real-time detection of signals, circumventing the need of analyzing samples by gel electrophoresis. Reactions were run on a MX3005P QPCR system (Agilent Technologies) and data were analyzed using the MxPro QPCR software (Agilent Technologies). For determination of gene copy number, reactions containing 10-fold serial dilutions of DNA with known concentration were included on each plate. This generated a standard curve to extrapolate gene copy numbers from the threshold cycle (Ct) numbers. Given that only 5% of the total DNA from each tick was analyzed in a reaction, the theoretic limit of detection was 20 copies of *B. burgdorferi* genome per tick.

### Rodent trapping in tiverton township

In September of 2010, rodent trapping was carried out in Tiverton Township for two consecutive nights. Sherman live traps (150 at the first night and 180 at the second night) were set using sunflower seeds and peanut butter sandwiches as bait. Captured rodents (3 from the first night and 7 from the second night) were euthanized by barbiturate overdose followed by heart exsanguination, and then checked for the presence of ticks. All 10 captured rodents were morphologically identified to be *P. leucopus* by John Harder, a mammalogist at The Ohio State University. Wildlife procedures were carried out in accordance with the Animal Welfare Act and were approved by ODH as part of disease surveillance investigation.

### Enzyme-linked immunosorbent assay (ELISA)

Serum samples were tested for IgM and IgG antibodies against *B. burgdorferi* antigens by enzyme-linked immunosorbent assay (ELISA). ELISA was performed in 96-well flat-bottom Immulon 1B plates (Thermo Scientific) according to a standard protocol (Hornbeck et al., 2001). Briefly, the plates were coated with 100 ng/well whole-cell lysate of *B. burgdorferi* type strain B31. Serum samples were assayed at a 1:400 dilution. The secondary antibodies, either goat anti-mouse IgM or IgG or goat anti-dog IgG, were conjugated with horseradish peroxidase (HRP) (Kirkegaard & Perry Laboratories) and used at a 1:1000 dilution. Signals were developed using the TMB Microwell Peroxidase Substrate System (Kirkegaard & Perry Laboratories) and measured using a SpectraMax M2 plate reader (Molecular Devices, LLC). For the study of *B. burgdorferi* seroprevalence in Ohio dogs, the titer of a highly positive serum sample was determined using the serial dilution method. Eight two-fold serial dilutions of this sample as well as eight blank wells were included on each ELISA plate, which were used for normalization to minimize plate-to-plate variation and for generating standard curves to extrapolate titers of unknown samples from the absorbance values.

### Immunoblot analysis

*Borrelia burgdorferi* whole cell lysate was separated on a sodium dodecyl sulfate-12% polyacrylamide gel prepared using the Mini-Protean Tetra hand cast system and a prep/2-D well comb (Bio-Rad Laboratories), and then transferred to a nitrocellulose membrane. Mouse serum samples were incubated at a 1:100 dilution with the membrane in a Mini-Protean II multiscreen apparatus (Bio-Rad Laboratories). After incubation with HRP-conjugated secondary antibodies, goat anti-mouse IgM or IgG, signals were developed using SuperSignal West Pico Chemiluminescent Substrate (Thermo Scientific) following manufacture's protocol, and captured on X-ray films.

### Virastripe® test

ViraStripe® Lineblot test kits (Viramed) received clearance from the Food and Drug Administration (FDA) in December 2009 for use in clinical diagnosis of human Lyme disease. The IgG Lineblot uses all 10 native *B. burgdorferi* B31 proteins recommended by the CDC for serological evaluation of human exposure to the Lyme disease spirochete. Serum samples from dogs and rodents were analyzed according to the manufacturer's protocol with only one modification—the alkaline phosphatase (AP)-conjugated secondary antibodies were goat anti-mouse IgM for the rodent samples and rabbit anti-dog IgG for the dog samples. Both of these conjugates were obtained from the Jackson ImmunoResearch Laboratories. The positive, cut-off, and negative controls were processed as instructed using the anti-human IgG-AP conjugate provided in the kit. Signals were developed using the chromogen/substrate solution provided in the kit, and reactions were stopped when the cut-off control was clearly visible. Based on the CDC recommendations, serum samples that reacted with at least 5 of the 10 proteins above the cut-off intensity were interpreted as positive.

### Statistical analysis

All statistical and graphic analyses were performed using the software GraphPad Prism 5 for Windows (version 5.0.1). All reported *P* values are 2-tailed. *P* values < 0.05 are considered statistically significant. The statistical test for each *P* value is indicated in the text.

## Results

### Evidence for the emergence of *I. scapularis* in Ohio

**Figure 1:**
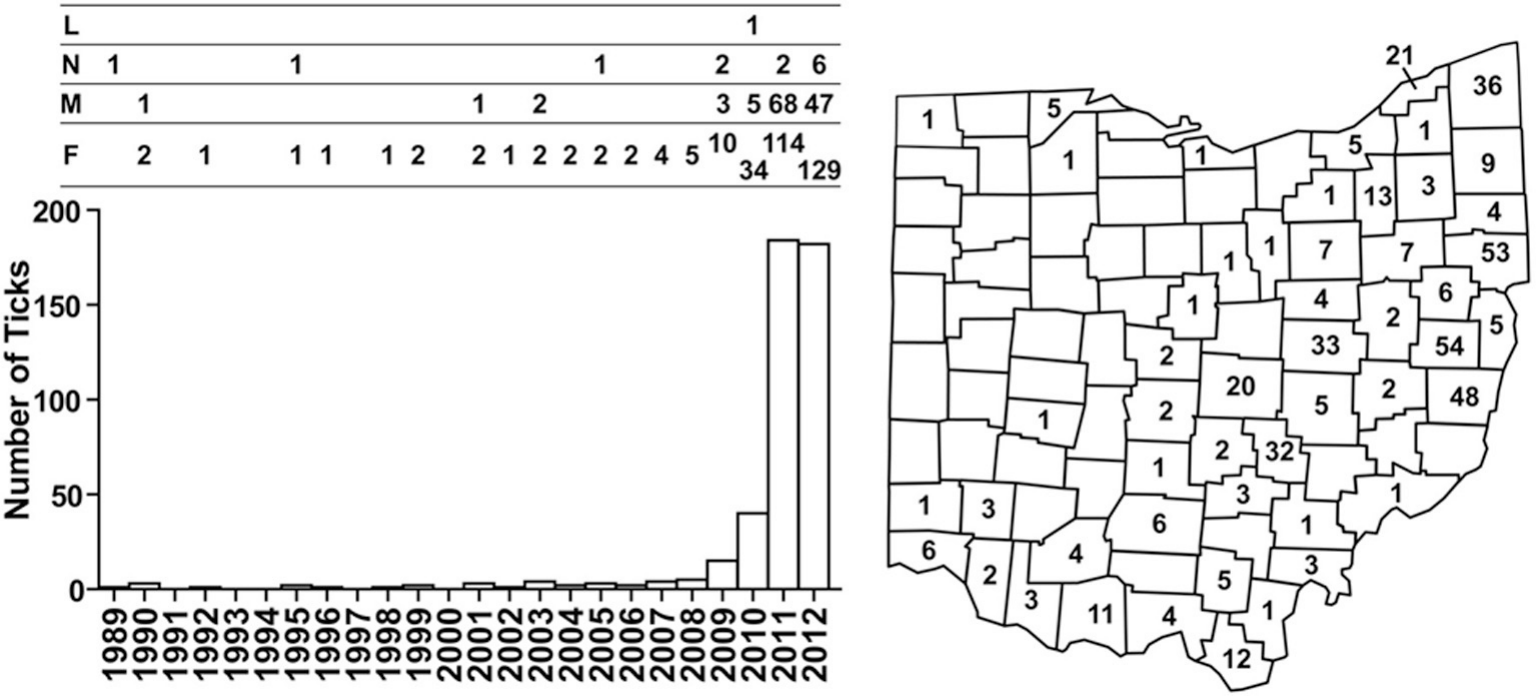
**Numbers and distribution of *I. scapularis* collected through the ODH tick surveillance program from 1989 to 2012**. Numbers of *I. scapularis* ticks are plotted according to year and the development stages of the ticks are indicated above the bar graph: L, larva; N, nymph; M, male, F, female. Cumulative numbers of *I. scapularis* found in Ohio counties are indicated on the map.

In 1989, a nymph collected in Butler County became the first *I. scapularis* confirmed in Ohio by the ODH tick surveillance program. The number of *I. scapularis* collected annually remained low from 1989 to 2008, averaging 1.75 (range, 0–5), accounting for well below 1% of ticks collected. Numbers of *I. scapularis* began to increase after 2008, with 15 in 2009, 40 in 2010, 184 in 2011, and 182 in 2012 (Figure 1). By 2012, *I. scapularis* ticks accounted for 24.8% of ticks collected by ODH tick surveillance program. Of the 456 *I. scapularis* collected 1989–2012, there were 315 (69%) female, 127 (28%) male, 13 (3%) nymphs, and 1 larva.

**Figure 2:**
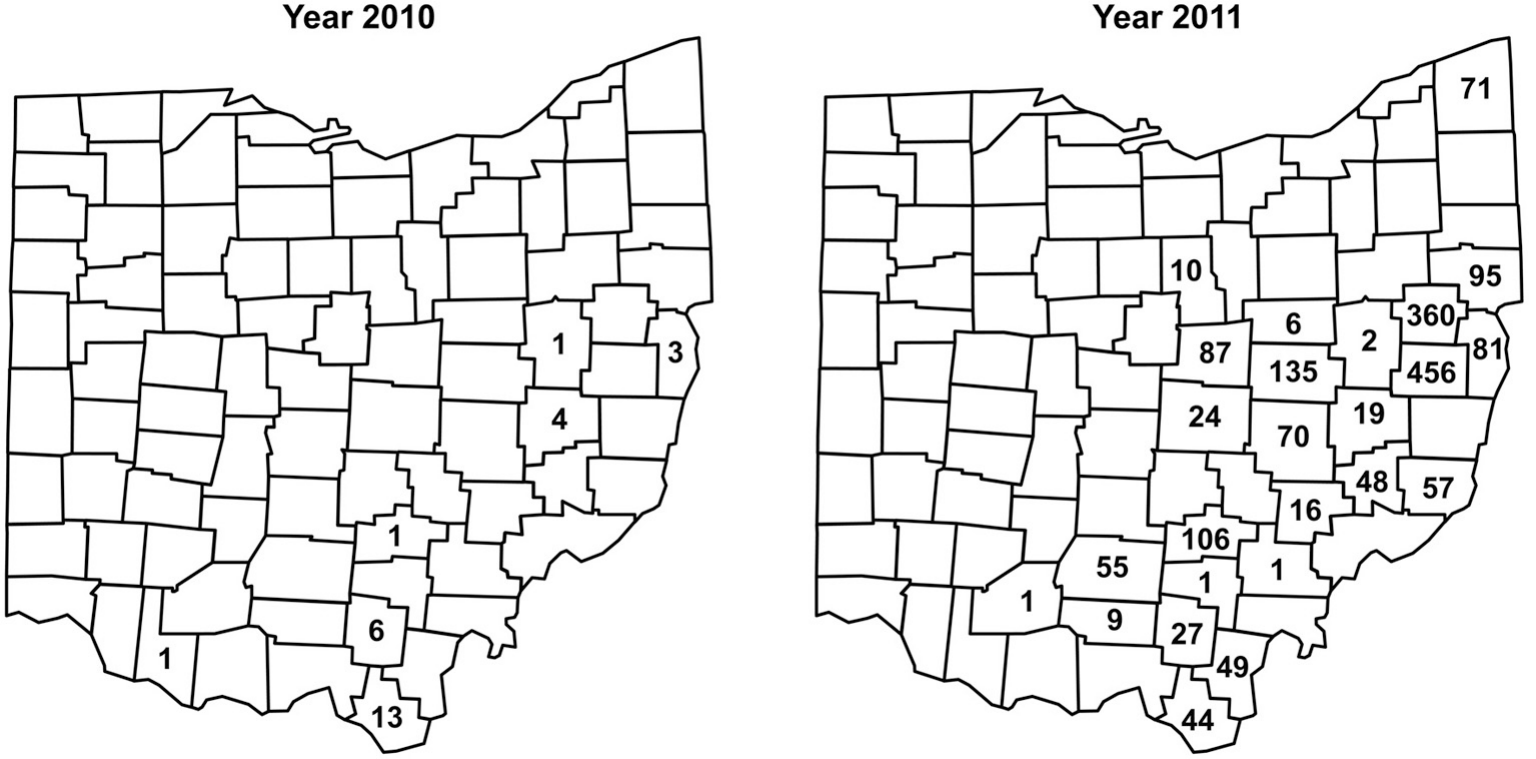
**Numbers and distribution of *I. scapularis* found on deer heads through the CWD surveillance program in 2010 and 2011**.

Between 2002 and 2008, no *I. scapularis* were found from examination of approximately 200–500 deer heads every year during the fall deer hunting season as part of the multi-agency surveillance of chronic waste disease (CWD) in Ohio. Almost all ticks found on deer heads during this period were *Dermacentor albipictus*, the one-host winter tick. In 2009, deer heads examined for CWD were not examined for ticks. In 2010, 29 *I. scapularis* were recovered from 12 (~6%) of approximately 200 deer heads examined. In 2011, 1,830 *I. scapurlaris* were recovered from 96 (17%) of 560 deer heads examined (Table 1). All 1,859 *I. scapularis* collected from deer heads were adults, 59% male and 41% female. The 29 ticks recovered in 2010 were from 7 counties, whereas the 1,830 ticks recovered in 2011 were from 25 counties (Figure 2). There was no noticeable increase in the number of *D. albipictus* found on deer heads in 2010 and 2011.

**Table 1:** ***Ixodes scapularis* found on deer heads during the fall deer hunting season**.

**Year**	**Number of deer heads examined for ticks**	**Number of deer heads with *I. scapularis***	**Number of *I. scapularis* (female/male)**	**Number of counties where *I. scapularis* found**
2010	~200	12	12/17	7
2011	560	96	747/1,083	25

Taken together, as of 2012, *I. scapularis* ticks were found in 57 of the 88 counties in Ohio (Figures 1, 2). The fact that these surveillance activities had been established for many years, and the increase in *I. scapularis* (both in numbers and in geographical ranges) was not observed until 2009 provides strong evidence that *I. scapularis* is an emerging vector in Ohio.

### Evidence for established *I. scapularis* colonies in tiverton township

On March 8, 2010, 10 adult *I. scapularis* were collected by flagging in less than 30 min. Between March and November of 2010, 207 adult and >200 larval *I. scapularis* were flagged from vegetation, and 52 nymphal and 14 adult *I. scapularis* were submitted by local residents who found and removed the ticks from people or domestic animals (Table 2). Therefore, all 3 active stages of *I. scapularis* have been found in Tiverton Township.

**Table 2:** **Tick survey at Tiverton Township in 2010**.

**Month**	**Number of *I. scapularis***			
	**Larvae**	**Nymphs**	**Adults**	
	**Vegetation**	**Host**	**Vegetation**	**Host**
March	–	–	27	–
April	–	–	70	–
May	–	6	43	3
June	–	46	–	4
July	>200	–	–	–
August	–	–	–	–
September	–	–	–	–
October	–	–	15	6
November	–	–	52	1

### Prevalence of *B. burgdorferi* infection in *I. scapularis* collected in Ohio

Nymphal and adult *I. scapularis* collected from Tiverton Township were analyzed by qPCR to determine the prevalence of *B. burgdorferi* infection. Larval *I. scapularis* ticks flagged from vegetation were not analyzed because transovarial transmission of *B. burgdorferi* is rare or nonexistent (Rollend et al., 2013). Initially, the *I. scapularis* ticks were analyzed individually. However, of the 27 adult *I. scapularis* flagged from vegetation in March 2010, none tested positive, indicating a low prevalence of *B. burgdorferi* infection. Thereafter, some *I. scapularis* ticks were tested in batches of 2–10 each. Overall, of the 273 *I. scapularis* tested, 138 were assayed individually and 135 were assayed in batches. Of the 15 *I. scapularis* samples tested positive for *B. burgdorferi flaB* DNA, one was a batch of 4 nymphs, one was a batch of 3 nymphs, and all the others were of single *I. scapularis*. To be conservative in our estimation of the infection rate, in both of these cases where a batch of multiple *I. scapularis* ticks tested positive, only one was considered positive.

**Figure 3:**
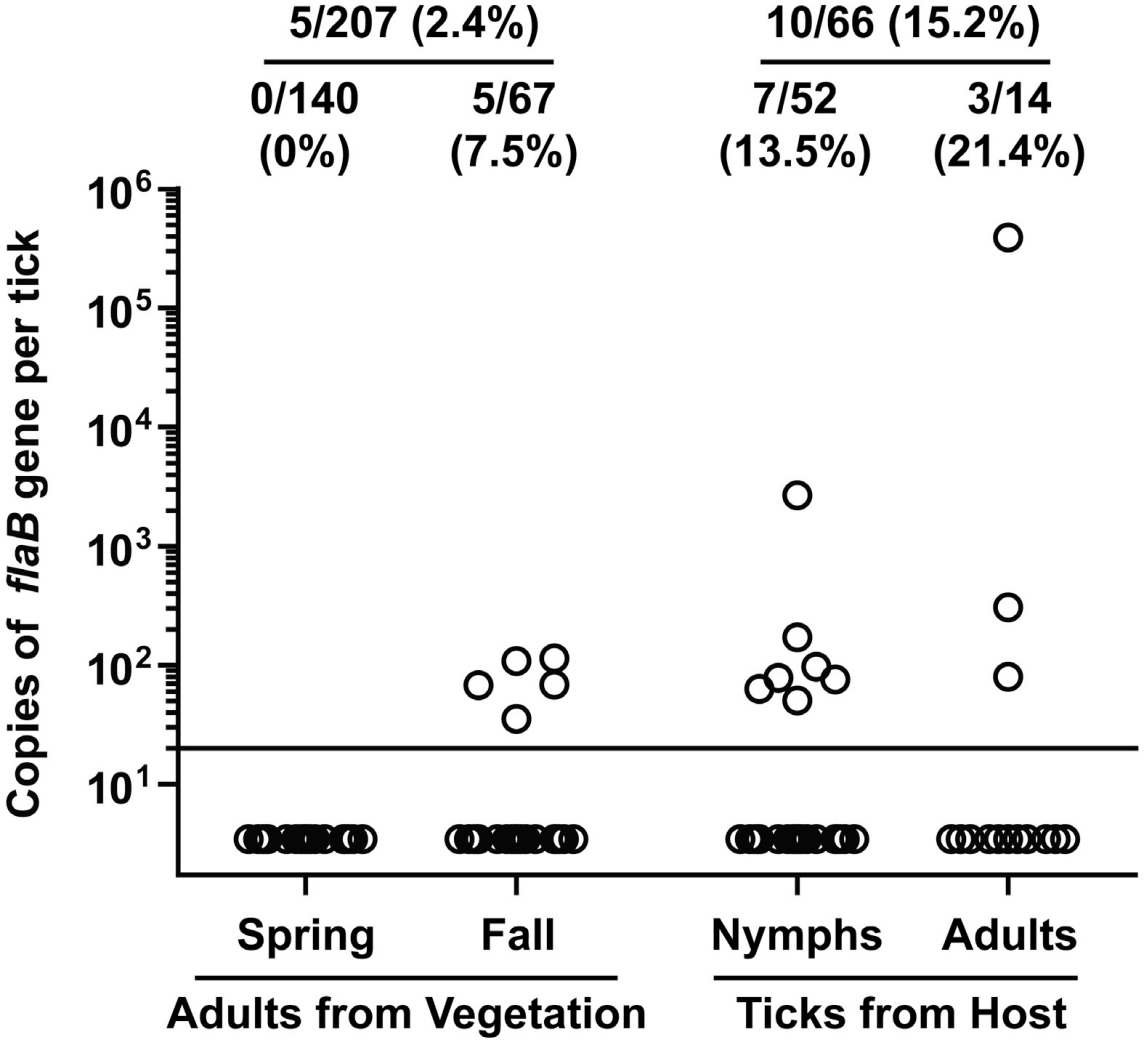
***Borrelia burgdorferi* DNA burden in *I. scapularis* collected from Tiverton Township**. Circles represent copies of the *B. burgdorferi flaB* gene in individual ticks. The ratio and percentage of samples tested positive were shown at the top of each group. The adult ticks collected from vegetation were divided into 2 groups depending on when they were collected, and the ticks collected from a host were divided into 2 groups according to their developmental stage.

Of the 207 adult *I. scapularis* flagged from vegetation, only 5 (2.4%) tested positive for *B. burgdorferi flaB* gene (Figure 3). Notably, none of the 140 adult *I. scapularis* collected from vegetation in spring tested positive, whereas 5 of the 67 (7.5%) adults collected in fall tested positive (*P* = 0.003, Fisher's exact test). Of the 66 *I. scapularis* collected from people or domestic animals, a total of 10 (15.2%) tested positive. The infection rate was 13.5% (7/52) for the nymphal *I. scapularis* and 21.4% (3/14) for the adult. In comparison, the infection rate in adult *I. scapularis* flagged from vegetation was significantly lower than that in adults collected from hosts (*P* = 0.0095, Fisher's exact test). Overall, 15 of 273 (5.5%) *I. scapularis* ticks were infected with *B. burgdorferi*, and the spirochete burden of infected *I. scapularis* ranged from 36 to 390,000 per specimen, with a median value of 80. The tick that had the highest burden was a male that was found crawling off a dog shortly after the removal of a partially engorged female from the animal. Similarly high levels of *Borrelia* burden in *Ixodes* species were previously reported by others (Wang et al., 2003; Wilhelmsson et al., 2010).

Although it is not a competent vector for transmitting *B. burgdorferi*, the dog tick *D. variabilis* can acquire the spirochete from infected rodents (Soares et al., 2006). Therefore, we also tested 67 adult *D. variabilis* flagged from vegetation in Tiverton Township, and 3 (4.5%) of them tested positive for *B. burgdorferi flaB* DNA. This indicated that the infection rate was similarly low in both *I. scapularis* and *D. variabilis* found in this area.

*Ixodes scapularis* from the ODH collections were also tested individually for the presence of *B. burgdorferi flaB* gene. Of the 184 *I. scapularis* ticks collected through ODH surveillance in 2011, only 27 were available for DNA testing. Among them, 2 (7.4%) tested positive, one with 220 and the other with 580 copies of *B. burgdorferi* genome per tick. Of the 1,830 *I. scapularis* recovered from CWD deer head surveillance in 2011, 220 (no more than 3 from each deer head) were selected for testing, and 15 (6.8%) tested positive, with the spirochete burden ranging from 111 to 11,800 per ticks. Therefore, the prevalence of *B. burgdorferi* infection in *I. scapularis* collected from Ohio statewide is similar to that of *I. scapularis* collected from Tiverton Township.

### *Borrelia burgdorferi* seroprevalence in field-captured *P. leucopus*

**Figure 4:**
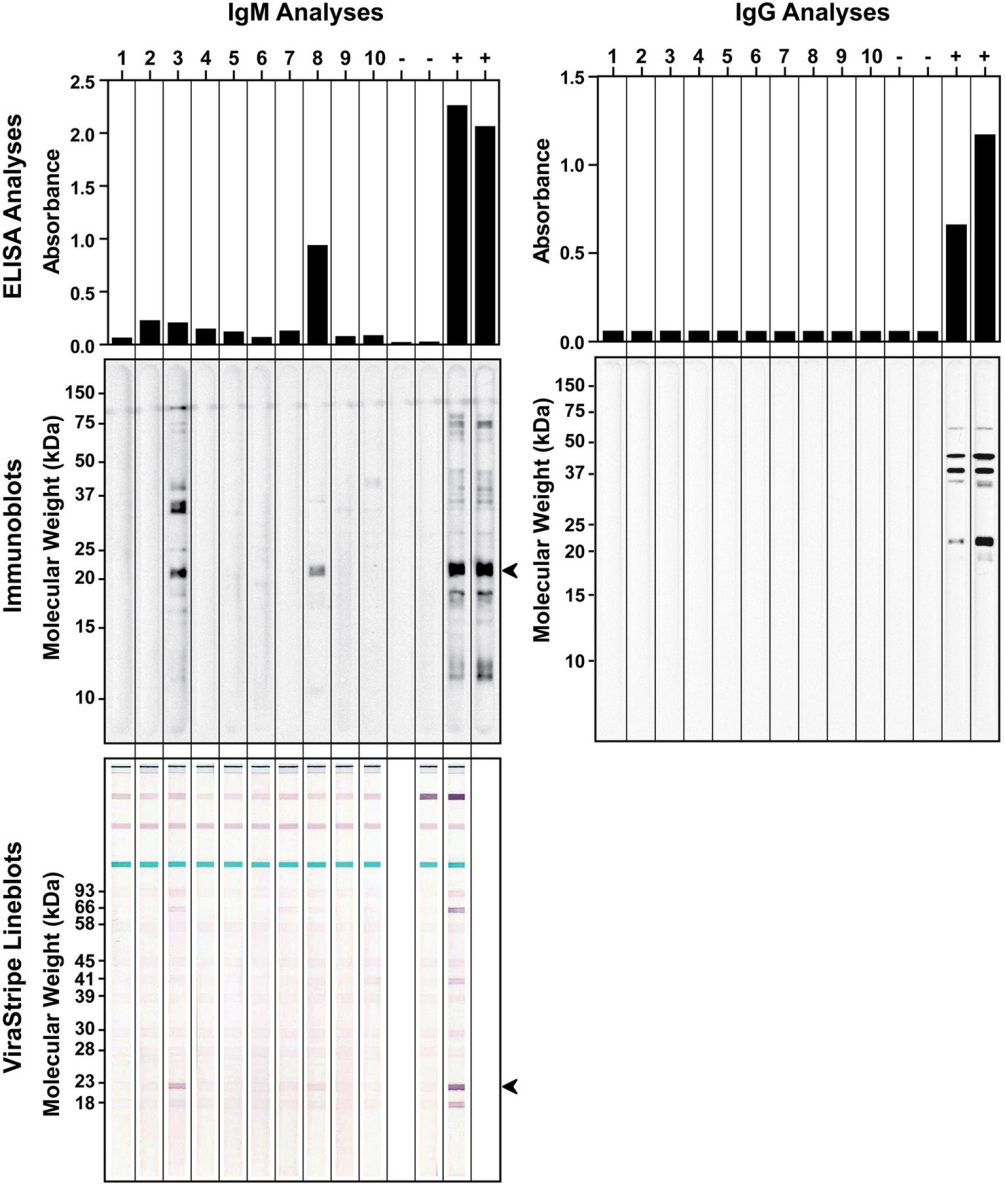
**Detection of antibodies to *B. burgdorferi* antigens in field-captured *P. leucopus* from Tiverton Township**. ELISA (top panels) and immunoblot (middle panels) analyses of IgM (left panels) and IgG (right panels) responses to *B. burgdorferi* whole-cell lysate were shown for serum samples from 10 captured *P. leucopus* and 4 laboratory mice that had been infested with naïve (−) or *B. burgdorferi*-infected (+) nymphs. The identity of the ~20-kDa protein, indicated by an arrowhead in the IgM immunoblot, was confirmed to be the 23-kDa OspC by the ViraStripe Lineblot analysis (bottom left panel).

We also investigated if the wild rodent population in Tiverton Township had been exposed to *B. burgdorferi*. In September of 2010, a total of 10 *P. leucopus* were captured during 2 consecutive nights. Serum samples were tested by ELISA and immunoblot analysis for IgG and IgM antibodies against *B. burgdorferi* antigens. For comparison, serum samples from laboratory C3H mice infested by naïve or *B. burgdorferi*-infected nymphs were included as negative and positive controls, respectively. All 10 *P. leucopus* serum samples tested negative for IgG antibodies to *B. burgdorferi*, but 2 of them (no. 3 and no. 8) had a positive IgM response by both ELISA and immunoblot analysis (Figure 4). It is intriguing that the no. 8 *P. leucopus* serum sample had much higher reactivity than the no. 3 *P. leucopus* serum sample in the ELISA assay, but in the immunoblot assay, the results appeared to be reversed, the no. 8 sample yielded much weaker signals than did the no. 3 sample. We repeated the IgM ELISA and immunoblot analyses for all samples to rule out the possibility that we inadvertently switched the samples. One possible explanation for this discrepancy may be that the *B. burgdorferi* component that reacted strongly with the no.8 serum sample in ELISA somehow was not efficiently separated by SDS-PAGE and transferred to the membrane in the immunoblot analysis. Nevertheless, serum samples from infected laboratory mice and the two positive *P. leucopus* serum samples all reacted very strongly with an approximately 20-kDa *Borrelia* antigen, which may be the outer surface protein C (OspC) that is highly expressed during mammalian infection. Thus, we further analyzed these serum samples using the ViraStripe IgG Lineblot, which contains 10 purified native proteins from *B. burgdorferi* B31 strain, including the 23-kDa OspC. The result indicated that the common band that reacted with both the laboratory positive controls and the two *P. leucopus* serum samples was indeed OspC (Figure 4).

The 10 captured *P. leucopus* were examined for ticks. Six mice had a total of 28 ticks attached, which included 23 larval *I. scapularis* as well as 2 nymphal and 3 larval *D. variabilis*. Only 1 of the 2 *D. variabilis* nymphs tested positive, which had 267 copies of *flaB* gene. The *D. variabilis* nymph that tested positive for *B. burgdorferi flaB* gene was collected from a rodent that tested negative for antibodies against *B. burgdorferi* antigen, suggesting that this dog tick may have acquired the spirochete when taking its first blood meal at the larval stage. One nymphal and 1 larval *D. variabilis* and 12 larval *I. scapularis* were found co-feeding on one of the rodents that tested positive for IgM antibodies to *B. burgdorferi*, but none of these ticks tested positive for *B. burgdorferi* DNA.

### *Borrelia burgdorferi* seroprevalence in Ohio dogs

**Figure 5:**
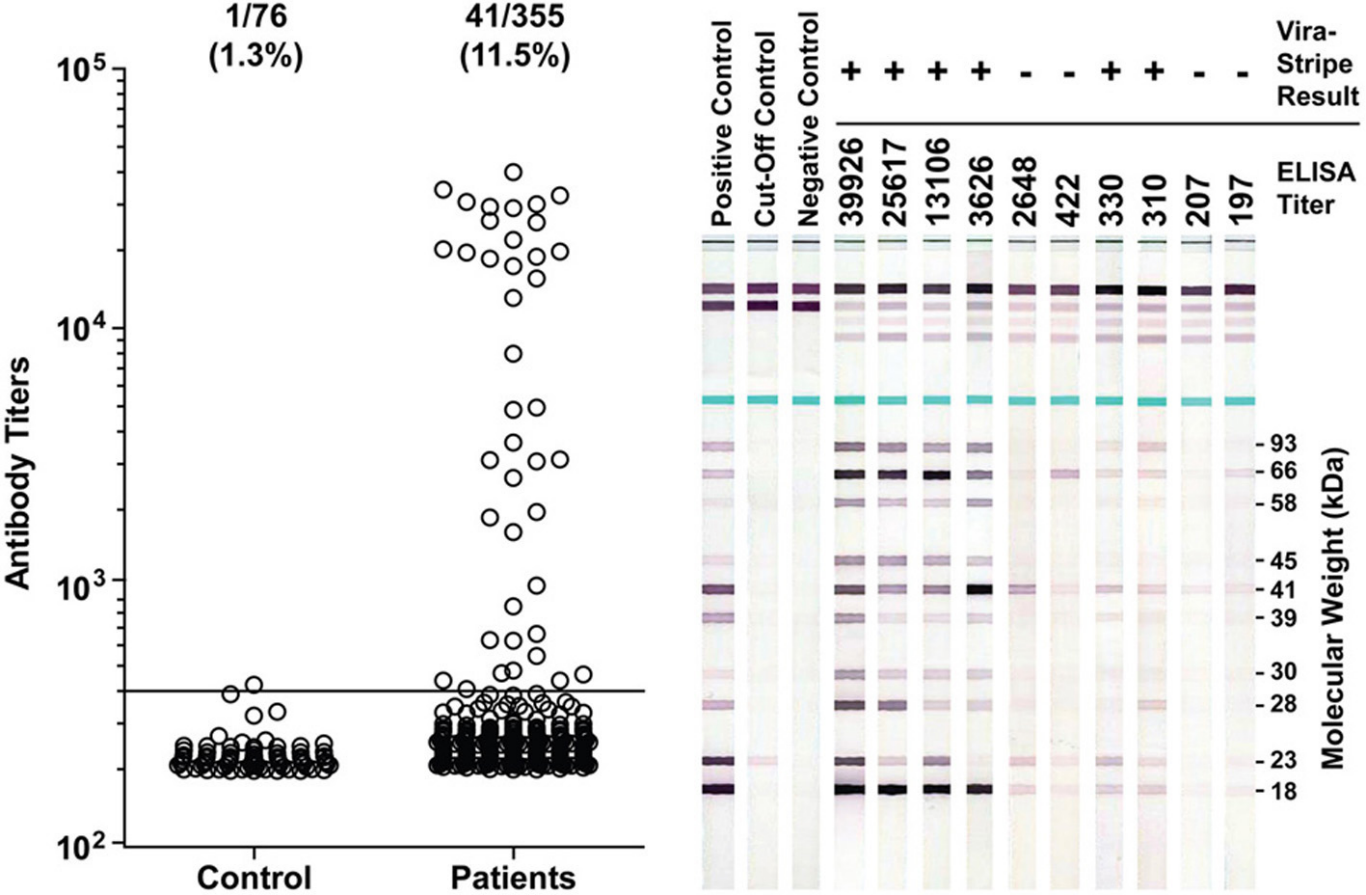
**Detection of antibodies to *B. burgdoferi* antigens in Ohio dogs**. Antibody titers as determined by ELISA are plotted in the left graph. The ratio and percentage of samples tested positive (titer >400) were shown above the graph for each group. The serum samples from 76 healthy greyhound blood donors were included as a control group for the 355 serum samples from canine patients who had blood work done at the OSU Veterinary Medical Center in summer of 2011. Representative ViraStripe Lineblots were shown on the right. The ELISA titer and the ViraStripe result for each test sample are indicated on the top.

It has been proposed that seroprevalence in dogs is a good indicator for Lyme disease risk (Bowman et al., 2009; Mead et al., 2011). Therefore, we investigated if there was evidence for *B. burgdorferi* exposure in Ohio dogs. From June to August of 2011, leftover plasma samples from a total of 355 Ohio dogs that had blood work done at the OSU Veterinary Medical Center were tested by ELISA for IgG antibodies to *B. burgdorferi* whole-cell lysate. Among them, 197 (55.4%) were from Franklin County, none were from Coshocton County, and the remaining 158 dogs were from 44 of the other 86 counties in Ohio. Serum samples from 76 healthy greyhound blood donors that had been screened to be free of common vector-borne diseases prior to enrollment and have since been treated with Frontline to prevent vector-borne diseases were included as a control group. The ELISA result indicated that the medium IgG titer was significantly higher in the patient group than in the control group (*P* < 0.0001, Mann-Whitney test) (Figure 5). With an arbitrary cut-off set at the titer of 400, the seroprevalence was 11.5% (41/355) for the patient group, significantly higher than the 1.3% (1/76) for the control group (*P* = 0.0045, Fisher's exact test). Of the 197 dogs from Franklin County, 26 (13.2%) tested positive, which is not significantly different from the 9.5% (15/158) for dogs outside of Franklin County (*P* = 0.4, Fisher's exact test).

We next compared the ELISA result with the ViraStripe Lineblot test, an FDA-approved test for diagnosis of human Lyme disease. A total of 71 samples were tested using the ViraStripe IgG Lineblot, which contains 10 purified native *B. burgdorferi* B31 proteins. Criteria for a positive ViraStripe result are based on the CDC recommendation—reactivity with no less than five of the ten proteins, and each at no less than the cut-off intensity. Notably, these criteria are more stringent and more specific than the criteria that we set for a positive ELISA result—reactivity with *B. burgdorferi* whole-cell lysate at a titer above 400. The 71 samples subjected to the ViraStripe test included all 42 ELISA-positive samples (41 patients and 1 greyhound donor) and 29 randomly selected ELISA-negative samples (20 patients and 9 greyhound blood donors). Results are shown in Table 3, and representative blots are shown in Figure 5. Twenty-seven (64%) of the 42 ELISA-positive samples tested positive by ViraStripe, whereas 2 (7%) of the 29 ELISA-negative samples tested positive by ViraStripe (*P* < 0.0001, Fisher's exact test). Moreover, of the ELISA-positive samples, the ones with a higher titer are more likely tested positive by ViraStripe than those with a lower titer. All of the 23 samples that had a titer ≥3,122 tested positive by ViraStripe whereas only 4 of the 19 samples with a titer between 400 and 3104 tested positive by ViraStripe (*P* < 0.0001, Fisher's exact test). Therefore, there is a strong correlation between the ELISA and the ViraStripe results (Table 3).

**Table 3:** **Comparison of ELISA and ViraStripe analyses of dog serum samples**.

**Sample ID^a^**	**ELISA Titer**	**ViraStripe Interpretation**	**Sample ID^a^**	**ELISA Titer**	**ViraStripe Interpretation**	**Sample ID^a^**	**ELISA Titer**	**ViraStripe**
POS001	39926	+	POS025	3064	+	NEG007	331	−
POS002	34205	+	POS026	2648	−	NEG008	330	+
POS003	32467	+	POS027	1960	+	NEG009	328	−
POS004	30652	+	POS028	1865	−	NEG010	325	−
POS005	30106	+	POS029	1640	−	NEG011	321	−
POS006	29401	+	POS030	1019	−	NEG012	313	−
POS007	29075	+	POS031	848	−	NEG013	310	+
POS008	25998	+	POS032	664	−	NEG014	305	−
POS009	25617	+	POS033	628	+	NEG015	258	−
POS010	21927	+	POS034	625	−	NEG016	229	−
POS011	20182	+	POS035	545	−	NEG017	207	−
POS012	19732	+	POS036	480	−	NEG018	205	−
POS013	19556	+	POS037	469	−	NEG019	204	−
POS014	18888	+	POS038	463	−	NEG020	204	−
POS015	18542	+	POS039	438	−	NEG021	203	−
POS016	17377	+	POS040	438	−	NEG022	201	−
POS017	15535	+	POS041	422	−	NEG023	200	−
POS018	13106	+	POS042	407	+	NEG024	199	−
POS019	7989	+	NEG001	389	−	NEG025	199	−
POS020	4950	+	NEG002	384	−	NEG026	197	−
POS021	4845	+	NEG003	350	−	NEG027	197	−
POS022	3626	+	NEG004	348	−	NEG028	197	−
POS023	3122	+	NEG005	336	−	NEG029	197	−
POS024	3104	−	NEG006	333	−			

a Of the 71 dog serum samples tested by ViraStripe, the 42 that had an ELISA titer above 400 were designated POS001-042, and the 29 that had an ELISA titer below 400 were designated NEG001-029.

## Discussion

Ohio had seen a low incidence of human Lyme disease, and this was largely attributed to the absence of the transmitting vector, *I. scapularis*. However, evidences presented in this study suggest that the blacklegged tick is becoming established in Ohio.

The most compelling evidences are from the tick surveillance programs that had been in place in Ohio for many years. First, the ODH tick surveillance program that was active 1983–2012 indicated that the number of *I. scapularis* found in Ohio had remained low until 2009, and had gone through an exponential increase in the following years. Second, the multi-agency CWD surveillance program that was active 2002–2011 also showed a sharp increase in the number of *I. scapularis* found on deer heads, from 29 in 2010 to 1830 in 2011. It is important to note that this increase is not due to invigorated surveillance. To the contrary, these data were recorded when both programs were being considered for termination due to decreasing budgets. The CWD monitoring program was terminated after the 2011 season. The ODH tick surveillance program, although active until the end of 2012, was scaled back in that final year, which may explain the plateau in the number of *I. scapularis* found. It is also important to point out the consistency between these data sets. During 2002–2008, while the ODH tick surveillance program recorded steady and low numbers of *I. scapularis*, no such ticks were recovered from deer heads. These data are also consistent with the result from a 2004–2007 survey by others that no nymphs were collected in and around Ohio (Diuk-Wasser et al., 2012). Therefore, the emergence of *I. scapularis* in Ohio appears to be relatively recent.

Of the 15 *I. scapularis* that were submitted to ODH in 2009, 3 (20%) were from Coshocton County. In addition, an Amish family in Tiverton Township of Coshocton County contacted Glen R. Needham regarding a case of un-reported human Lyme disease and sightings of *I. scapularis* on their property. Glen R. Needham thus surveyed this area in Coshocton County to determine if *I. scapularis* populations were established. The seasonal activities of larval, nymphal, and adult *I. scapularis* in Tiverton Township as shown in Table 2 were consistent with those described for other endemic areas in the US (Fish, 1993), with adults being active in spring and fall, nymphs being active in early summer, and larvae being active in summer. Finding all 3 active stages of *I. scapularis* in Tiverton Township demonstrated the presence of established tick populations in this area, although it did not provide a timeline for the establishment. The low rate of *B. burgdorferi* infection in the *I. scapularis* and *D. variabilis* ticks found in this area and in the field-captured *P. leucopus*, however, is more consistent with a recent emergence rather than a long-term establishment of the enzootic life cycle in the area.

Given that enzootic life cycle *B. burgdorferi* in Ohio appears to be nascent, we expect that the number of *I. scapularis* and the percentage of *B. burgdorferi*-infected *I. scapularis* found in this state will continue to increase. However, we do not expect to see further expansion of the range of this tick, because the distribution of *I. scapularis* found in Ohio so far is, by and large, consistent with the state's deciduous forest range. Given the proximity of these Ohio counties to highly endemic Pennsylvania, one possibility is that the emergence of this tick vector in Ohio simply reflects the continuing expansion of the Northeast Lyme disease-endemic region in the US. Migrating birds and/or deer as well as human activities could play a role in the spreading of the Lyme disease vector and agent. For example, transporting harvested deer from tick-infested areas of nearby Pennsylvania may be a factor because hides are often composted in wooded areas where ticks could detach and begin a “hot-spot.” Hunters should be alerted to this possibility so that hides can be properly disposed.

The apparently recent emergence of *I. scapularis* in Ohio is expected to lead to higher incidences of Lyme disease in people and in domestic animals in the future. Of 355 Ohio dog serum samples that were tested by ELISA, 41 (11.5%) had a titer above 400, and 27 (66%) of these 41 ELISA-positive samples tested positive by an immunoblot analysis using the CDC criteria set for human exposure to the Lyme disease spirochete. Given that the SNAP 4Dx Plus Test (IDEXX) employs a more stringent criterion for exposure to the Lyme disease spirochete—the presence of antibodies to a specific 26-amino acid residue peptide known as C6 (Liang et al., 1999), not all of the 27 ELISA-positive and ViraStripe-positive samples are expected to test positive by the SNAP 4Dx Plus Test. Also, given that these dogs were seen at a major medical center, the rate of seroprevalence in this group of patients could be higher than that in the general population. According to data published by the Companion Animal Parasite Council website (www.capcvet.org), the percentage of Ohio dogs tested positive for exposure to the Lyme disease spirochete by the SNAP 4Dx Plus Test was 0.39% (218 of 54,963) for 2011, 0.55% (250 of 45,376) for 2012, 0.56% for 2013 (543 of 96,016), and 0.62% (102 of 16,343) for 2014 as of April 23, 2014, showing a significant trend of increase (*P* < 0.0001, Chi-square test for trend). According to data available from the CDC, the number of confirmed human Lyme disease cases in Ohio was 21 (0.2 per 100,000 persons) for 2010, 36 (0.3 per 100,000 persons) for 2011, 49 (0.4 per 100,000 persons) for 2012, and 74 (0.6 per 100,000 persons) for 2013. However, preliminary estimates presented by CDC at the 2013 International Conference on Lyme Borreliosis and Other Tick-Borne Diseases suggest that the actual number of Lyme disease cases may be 10 times higher than the number of reported cases. Given that Ohio was not previously considered endemic for Lyme disease, it is likely that many cases in Ohio might have gone unreported or even undetected. Nevertheless, these numbers of confirmed Lyme disease cases in Ohio, again, indicate a significant trend of increase (*P* < 0.0001, Chi-square test for trend). The increased exposure to the Lyme disease agent seen in people and dogs in Ohio further corroborate with our data, and is consistent with a recent emergence of *I. scapularis* in this state. Healthcare professionals (e.g., physicians, nurse practitioners, and veterinarians) and residents in Ohio should become aware of this emerging tick and its associated risk of disease.

## Author contributions

Glen R. Needham, Kathleen A. Smith, Richard E. Gary, and Xin Li. designed the experiments, Peng Wang and Meaghan N. Glowacki performed laboratory analysis, and all authors contributed to data analysis and interpretation. Peng Wang, Meaghan N. Glowacki and Xin Li wrote the manuscript, and Armando E. Hoet, Glen R. Needham, Kathleen A. Smith, and Richard E. Gary critically revised the manuscript. All authors approved the final manuscript and agreed to be accountable for all aspects of the work in ensuring that questions related to the accuracy or integrity of any part of the work are appropriately investigated and resolved.

### Conflict of interest statement

The authors declare that the research was conducted in the absence of any commercial or financial relationships that could be construed as a potential conflict of interest.
